# A novel 1.38-kb deletion combined with a single nucleotide variant in *KIAA0586* as a cause of Joubert syndrome

**DOI:** 10.1186/s12920-023-01438-6

**Published:** 2023-01-12

**Authors:** Yue Shen, Chao Lu, Tingting Cheng, Zongfu Cao, Cuixia Chen, Xu Ma, Huafang Gao, Minna Luo

**Affiliations:** National Human Genetic Resources Center, National Research Institute for Family Planning, Beijing, China

**Keywords:** Joubert syndrome, *KIAA0586*, Variant, Genomic deletion

## Abstract

**Background:**

*KIAA0586*, also known as *Talpid3*, plays critical roles in primary cilia formation and hedgehog signaling in humans. Variants in *KIAA0586* could cause some different ciliopathies, including Joubert syndrome (JBTS), which is a clinically and genetically heterogeneous group of autosomal recessive neurological disorders.

**Methods and Results:**

A 9-month-old girl was diagnosed as JBTS by the “molar tooth sign” of the mid-brain and global developmental delay. By whole-exome sequencing, we identified a single nucleotide variant c.3303G > A and a 1.38-kb deletion in *KIAA0586* in the proband. These two variants of *KIAA0586* were consistent with the mode of autosomal recessive inheritance in the family, which was verified using Sanger sequencing.

**Conclusions:**

This finding of a compound heterozygote with a 1.38-kb deletion and c.3303G > A gave a precise genetic diagnosis for the patient, and the novel 1.38-kb deletion also expanded the pathogenic variation spectrum of JBTS caused by *KIAA0586*.

**Supplementary Information:**

The online version contains supplementary material available at 10.1186/s12920-023-01438-6.

## Introduction

JBTS (JBTS, OMIM#PS213300) is a clinically and genetically heterogeneous group of autosomal recessive neurological disorders characterized by a distinctive mid-hindbrain malformation (“molar tooth sign” on brain imaging), hypotonia and developmental delay/intellectual disability [1–3]. *KIAA0586* (also known as *Talpid3*) encodes a centrosomal protein that is essential for primary cilia formation and hedgehog signaling [4–6]. Variations in *KIAA0586* could cause some different ciliopathies, such as one subtype of Joubert syndrome (JBTS23, OMIM #616,490), preaxial polydactyly and short rib thoracic dysplasia 14 (SRTD14; OMIM #616,546) [7–10]. More than 50 variations in the *KIAA0586* gene were reported to be cause of JBTS in homozygous or compound heterozygous states, and the types of these variations varied, including missense/nonsense mutations, splice sites, small insertions/deletions or long-fragment genomic deletions [7, 11–17].

With whole-exome sequencing (WES), we identified a novel 1.38-kb deletion and a single nucleotide variant (SNV) in *KIAA0586* in a Joubert syndrome patient and confirmed them using Sanger sequencing in the family.

## Materials and methods

### Clinical summary

**Fig. 1:**
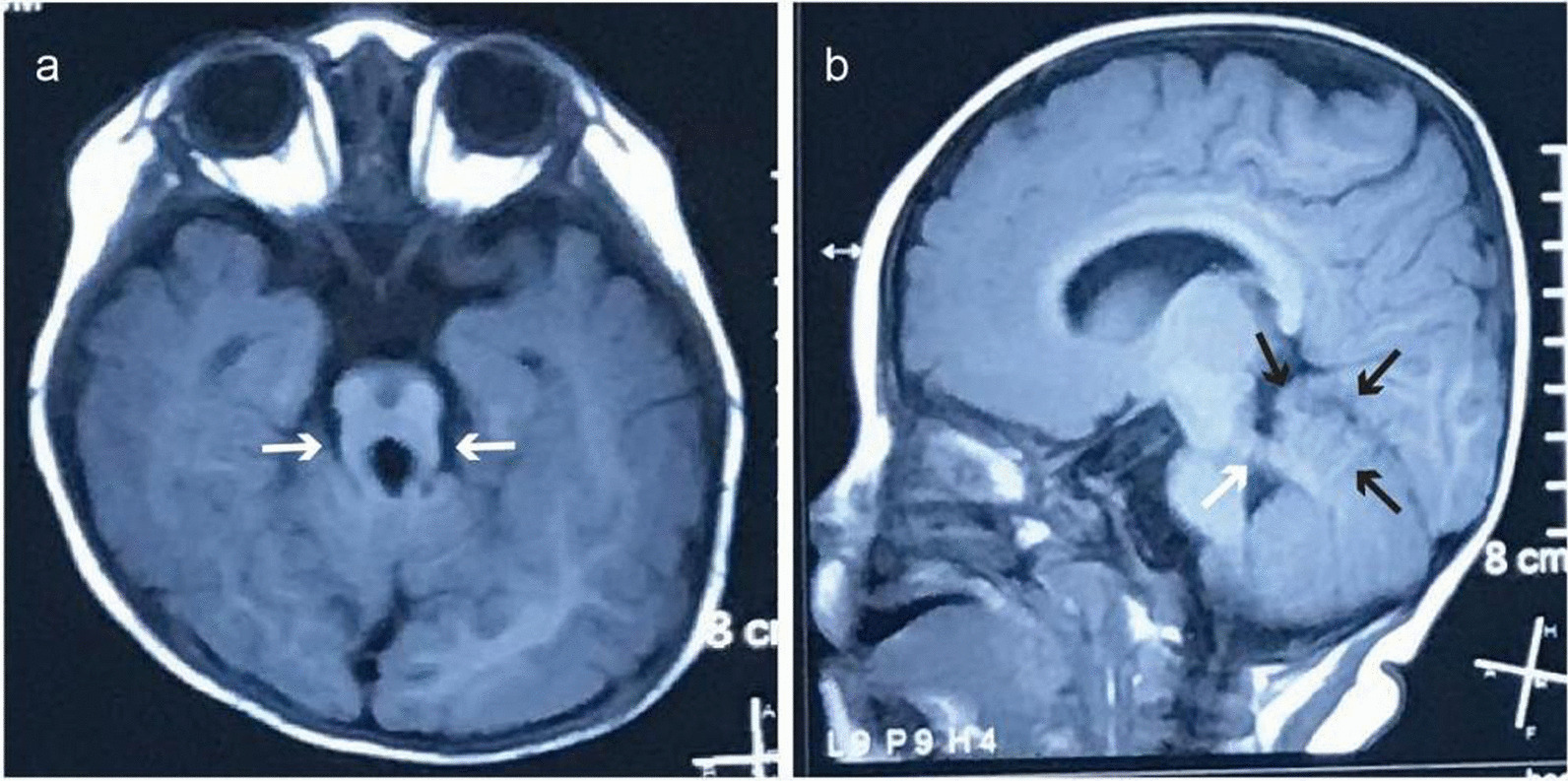
Brain magnetic resonance imaging (MRI) findings for the Joubert syndrome individual. Molar tooth sign with lengthening and thickening of superior cerebellar peduncles as indicated by white arrows in **a** and **b**, and the shape of cerebellar vermis was irregular and partially missing as indicated by black arrows in b

A 9-month-old girl was born after normal labor with a birth weight of 3.30 kg and height of 50 cm at 39 weeks gestation. Her parents were inconsanguineous. She is the second child of her family and has an elder sister. Her parents and sister are unaffected. The girl was hospitalized because of severe respiratory problems at birth, and then for delayed global development at 9 months. She cannot raise her head until 4 months, cannot turn over until 8 months, cannot sat all by herself, and cannot stand when supporting station. She can grasp objects on her own initiative. She can identify her mother and speak with only monosyllables, i. e. mama, baba. She often stuck out her tongue unconsciously. The girl had moderate intellectual disability, and severe retardation for large motor abilities, as evaluated by the Chinese Developmental Scale for children aged 0–6 years (WS/T 580–2017). The girl was fed with milk powder. The girl had a height of 74 cm (+ 1 SD), weight of 9 kg (median ~  + 1 SD), and occipitofrontal circumference of 45 cm (+ 1 SD) at 9 months old. Physical examination showed strabismus, hypotonia and lower myodynamia. No abnormalities were mentioned for the heart, liver, gallbladder, spleen, kidneys, ureter, or bladder. Magnetic resonance imaging of her brain revealed that the superior cerebellar peduncles were thickened and lengthened (molar tooth sign), and the shape of the cerebellar vermis was irregular and partially missing (Fig. 1). There was a link near the midline of the bilateral cerebellar hemispheres. The upper part of the fourth ventricle seemed to be bat wings. Thus, the girl was diagnosed with Joubert syndrome.

### Genetic investigations

A 2 mL EDTA anticoagulant venous blood sample was obtained from the proband, her parents and sister. Genomic DNA was extracted from whole blood using QIAamp^®^ DNA Blood mini Kit (QIAGEN, Germany) according to the manufacturer’s protocol. Whole-Exome Sequencing (WES) and SNVs/InDel analysis were performed for the proband as previously described [18]. Splice variants were evaluated using the online tool SpliceAI (https://spliceailookup.broadinstitute.org). Identification and annotation of genetically mobile domains and the analysis of domain architectures were performed by SMART (a Simple Modular Architecture Research Tool) (http://smart.embl-heidelberg.de/).

The WES data were further analyzed for copy number variants (CNVs). We made a virtual gene panel for JBTS and other ciliopathies using Phenolyzer [19] and OMIM (https://omim.org/) (There were a total of 169 related genes, data not shown). We obtained the chromosomal position information of exons of the classical transcripts from UCSC (https://genome.ucsc.edu/) for all candidate genes, and generated a bed file for all exons. Based on the hypothesis that exon deletion/duplication rarely occurs in JBTS, we performed CNV assessment using a read depth-based CNV detection algorithm [20]. Three WES data at the same sequencing batch, including the proband and two unaffected individuals were involved in CNV analysis. The mean depth of each exon was obtained with Samtools (https://anaconda.org/bioconda/samtools). Using Integrative Genomics Viewer (IGV, http://software.broadinstitute.org/software/igv/), the called exon deletions/duplications were confirmed manually.

### SNV verification

PCR was performed using specific primer pairs for candidate SNV validation screened from WES in the proband and her family members. The Additional file 1: Table S1 lists the primer sequences.

### CNV verification and precise breakpoint determination

To verify the CNV, we firstly carried out a relative quantitative PCR to analyze genome relative copy number of exon 15 of *KIAA0586* (NM_001244189.1) in the family. Triplicate quantitative PCR for genomic DNA was performed using SYBR Green qPCR Master Mix (Life Technologies, USA) on ABI7500 (Life Technologies, USA). *ALB* (albumin) was used as an internal control. The delta-delta CT value analysis method was used to evaluate the relative copy number of genome *KIAA0586* exon 15 for all samples. Target and internal control gene primer pairs are listed in Additional file 1: Table S1. Then, we performed a long PCR to determine the precise breakpoint of the deletion. Based on the predicted PCR products length, genomic DNA was amplified following the long PCR program: 94 °Cfor 3 min; 98 °Cfor 10 s, 68 °Cfor 3 min (30 cycles); 72 °Cfor 10 min. The long PCR primer sequences are shown in Additional file 1: Table S1.

### RNA extraction and reverse transcription

The Tempus™ Spin RNA Isolation Kit (Invitrogen, USA) was used for RNA extraction from whole blood cells of the proband and her parents and sister. One microgram RNA was reverse transcribed into cDNA using a SuperScript™ IV First-Strand Synthesis System Kit (Invitrogen, USA). The primer sequences for investigating the effect of deletion in the transcript are listed in Additional file 1: Table S1.

The PCR amplification products from the verification of SNVs and CNVs were analyzed by agarose gel electrophoresis, and then Sanger sequencing was performed on an ABI3730xl Genetic Analyzer (Life Technologies, USA) following the manufacturer's protocol.

## Results

**Fig. 2:**
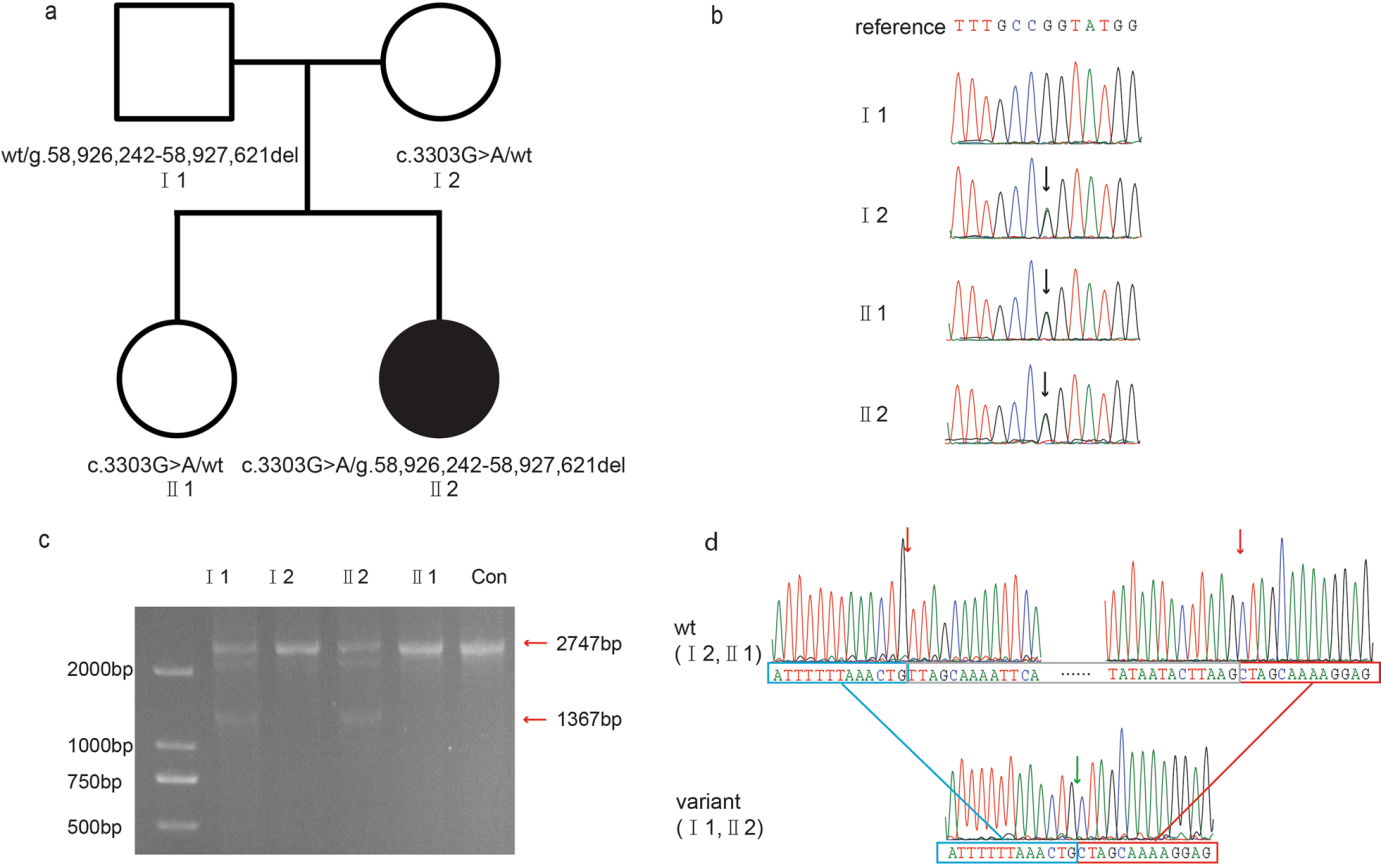
Pedigree of Joubert syndrome patient and Sanger chromatograms. **a** Pedigree of the Joubert syndrome patient. **b** Sanger sequencing of *KIAA0586* variant c.3303G > A. The black arrow indicates the position of variant. **c** Gel image for long PCR. Two fragments were obtained (2,747 bp and 1,367 bp) from the patient (II 2) and the father (I 1). **d** Sanger sequencing of *KIAA0586* genomic deletion g.58,926,242–58,927,621del. Red arrow indicates the position of the break locus, and green arrow indicates the position of the connection locus

**Fig. 3:**
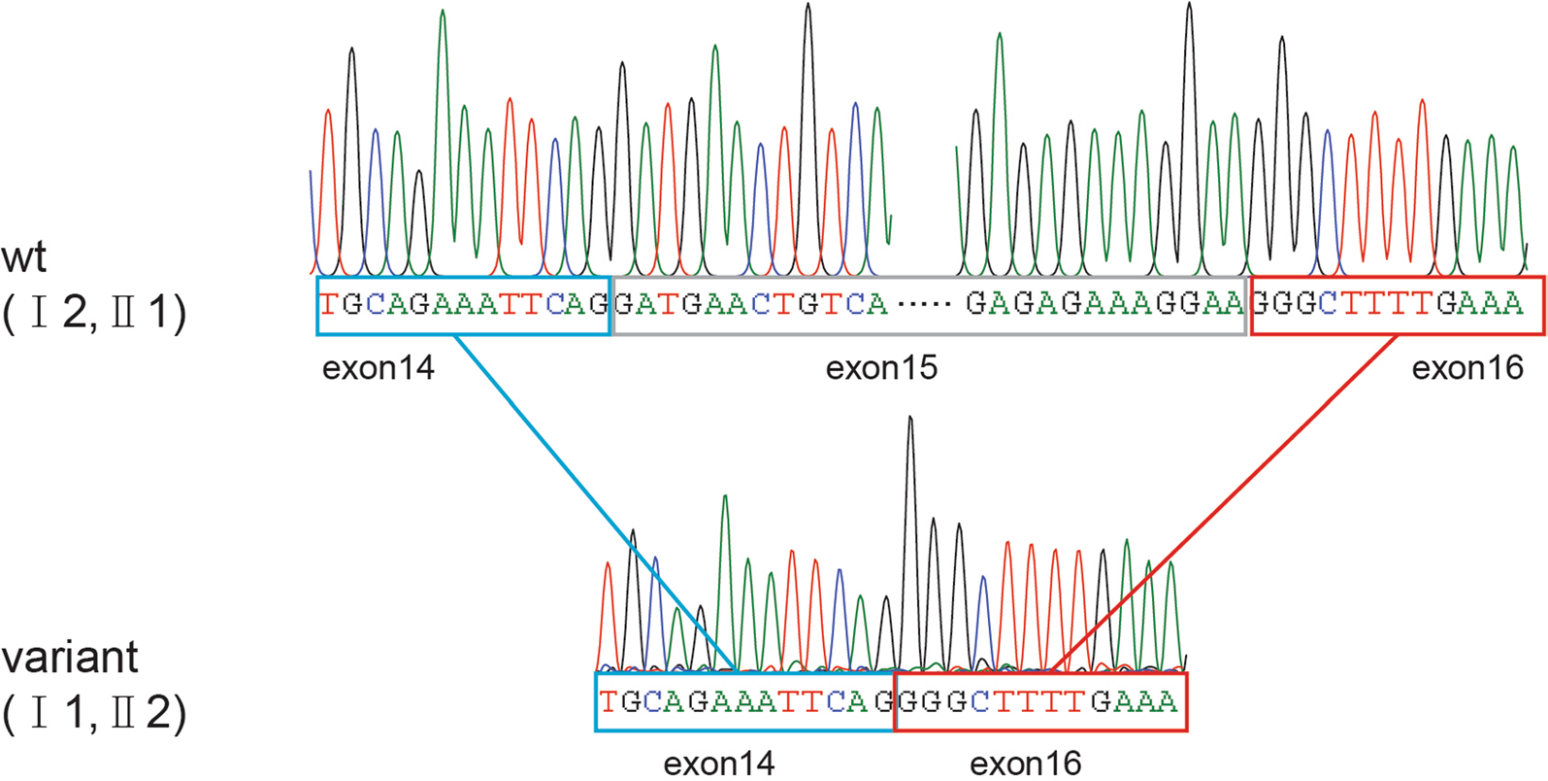
Sanger sequencing for the splicing effect of g.58,926,242–58,927,621del in the transcript. Exon 15 was skipped in the transcript for the patient (II 2) and the father (I 1)

WES revealed a splicing variant (NM_001244189.1: c.3303G > A) and a genomic region with a 1.38-kb deletion (NC_000014.9: g.58,926,242–58,927,621del) in *KIAA0586* as causes of Joubert syndrome in a 9-month-old girl (Fig. 2). The reported splicing variant c.3303G > A (NM_001244189.1) was verified in the heterozygous state in the proband, her mother and sister, which was validated by direct Sanger sequencing (Fig. 2 b). This variant might cause aberrant splicing predicted by the online software SpliceAI, remove 56 base pairs at the end of exon 23 (NM_001244189.1) and terminate early translation (NP_001231118.1: p.Pro1084LysfsTer22), which was described in detail by Bachmann-Gagescu et al. [7]. Our RNA extraction and reverse transcription experiments also confirmed this finding (data not shown). By long PCR, there were two fragments amplified on the patient and her father, only one fragment on the mother and sister (Fig. 2c and Additional file 3: Fig. S1). The precise breakpoints of the deletion were at positions 58,926,242 and 58,927,621 on chromosome 14 determined by Sanger sequencing (Fig. 2d). The 1.38-kb deletion led to the removal of part of intron 14, exon 15 and part of intron 15 of *KIAA0586* (NM_001244189.1), causing exon 15 skipping in the transcript (Fig. 3), which was predicted to be a 76 amino acid deletion (position 605 to 681) encoded by exon 15 during protein translation. Compared to the mother and the sister, results of qPCR showed that the proband and her father only had relative half-fold copy for exon 15 (Additional file 2: Table S2). The Database of Genomic Variants (http://dgv.tcag.ca) and Decipher (https://decipher.sanger.ac.uk) had not previously described deletions for this genomic region. According to the ACMG guidelines, these two variants were classified as pathogenic and likely pathogenic. No additional deleterious variant of other Joubert syndrome- or ciliopathy-related genes was identified in the WES data of the proband.

## Discussion

We identified a novel genomic deletion (g.58,926,242–58,927,621del) in the *KIAA0586* gene combined with a splicing variant (NM_001244189.1: c.3303G > A) as a cause for JBTS. The deletion was paternally inherited, and the c.3303G > A variant was maternally inherited. These two variants were present in the heterozygous state in the affected child, which was consistent with the autosomal recessive inheritance mode.

DNA fragments of approximately > 50 bp are defined as CNVs, which include deletions and duplications [21, 22]. In addition to SNVs or small insertions/deletions, CNVs may also be the cause of some human diseases [23, 24]. Three different long fragment genomic deletions in *KIAA0586* were reported previously [13–15]. With a functional genome-wide siRNA screen, Roosing et al*.* found a > 15.6 kb deletion spanning exons 12 to 20 (NM_001244189.1, chr14:?_58923420_58938997_?del; c.1413-?_2793 + ?del) in a Turkish patient, and the breakpoints were not defined[14]. In the transcript, this deletion was predicted to lead to the removal of exons 12 to 20 in *KIAA0586* (NM_001244189.1), causing direct splicing of exons 11 to 21, leading to early translational termination (p.Phe472LysfsTer49). An 8.26-kb genomic deletion that led to the removal of exons 8, 9 and 10 was reported by Malicdan et al. in two Jeune and Joubert syndrome families [15]. This deletion removed a 544-bp gene fragment including exons 8, 9 and 10 in *KIAA0586* (NM_001244189.1) in the transcript, and led to a frameshift and formation of a premature stop codon (p.Val249GlufsTer3) in exon 11. Sumathipala et al. reported another 8.3 kb genomic deletion in *KIAA0586* using WGS data [13]. The deletion also led to the removal of exons 8, 9 and 10 and then made an early termination (p.Val249GlufsTer3) in the transcript. However, these two deletions had different breakpoints, the former was in chr14 (GRCh37):g.58,910,322–58,918,560, and the latter in chr14 (GRCh37):g.58,910,301–58,918,610. In humans, TALPID3 domain is located at amino acids 183 to 1,411 as the critical functional domain of KIAA0586, which spans exon 6 to 30. The reported *KIAA0586* long genomic deletions were all located in this domain and predicted to make truncated proteins. The other single nucleotide variants (SNVs)/indels in these affected individuals also produce premature stop codon. The novel genomic deletion (g.58,926,242–58,927,621del) in our study removed exon 15 in *KIAA0586* (NM_001244189.1), and divided the whole TALPID3 domain into two parts (amino acids 183 to 608 and 605 to 1335), which suggested that the changed protein could perform incomplete TALPID3 functions. The other variant (c.3303G > A, p.Pro1084LysfsTer22) produced truncated protein, which may be the reason why the patient's phenotype was milder than those previously reported. Further exploration of *KIAA0586* function is still necessary.

Compared to SRTD14 and other forms of Joubert syndrome, the phenotype of JBTS23 is relatively mild, and most organ systems are generally unaffected. We diagnosed the 9-month-old girl with Joubert syndrome basically for hindbrain malformation and other characteristics according to the criteria of JBTS diagnosis. The other symptoms in our patient included breathing problems, strabismus, hypotonia and lower myodynamia, but no abnormalities for other organ systems. We identified pathogenic compound heterozygous variants in *KIAA0586* by WES, which was compatible with the reported genotype–phenotype for JBTS caused by *KIAA0586*. Thus, a genetic diagnosis of JBTS23 was given to the girl. Abnormal neurological, skeletal, and renal phenotypes are often observed in JBTS patients with *KIAA0586* variants [7]. This suggests it is necessary to pay attention to the development of these systems in medical examinations for this girl.

In our study, the finding of a compound heterozygote with a 1.38-kb deletion and c.3303G > A variant gave a precise molecular diagnosis for the Joubert syndrome patient, and was helpful for the doctors and parents providing quality care to her. Meanwhile, the novel 1.38-kb deletion also expanded pathogenic variation spectrum of JBTS caused by *KIAA0586*. Further functional validation is still necessary for clarify of the pathogenic mechanism of the *KIAA0586* gene in Joubert syndrome.

## Supplementary Information

**Additional file 1.  Table S1.** Primers for verification of candidate variants.

**Additional file 2.  Table S2.** Results of qPCR for the 1.38kb deletion.

**Additional file 3.  Fig. S1.** Original gel image for long PCR.

## Data Availability

The datasets generated and/or analysed during the current study are available in the human reference 8 genome (GRCh37/hg19, http://genome.ucsc.edu), NM_001244189.1 (https://www.ncbi.nlm.nih.gov/nuccore/NM_001244189.1).
