# Supplementary material for: A novel 1.38-kb deletion combined with a single nucleotide variant in KIAA0586 as a cause of Joubert syndrome
Source: BMC Med Genomics. 2023 Jan 12;16:4. doi: 10.1186/s12920-023-01438-6 (PMC9838056; doi:10.1186/s12920-023-01438-6)
Supplement: Supplementary file 3 — Additional file 3. Fig. S1. Original gel image for long PCR. [file 12920_2023_1438_MOESM3_ESM.pdf]

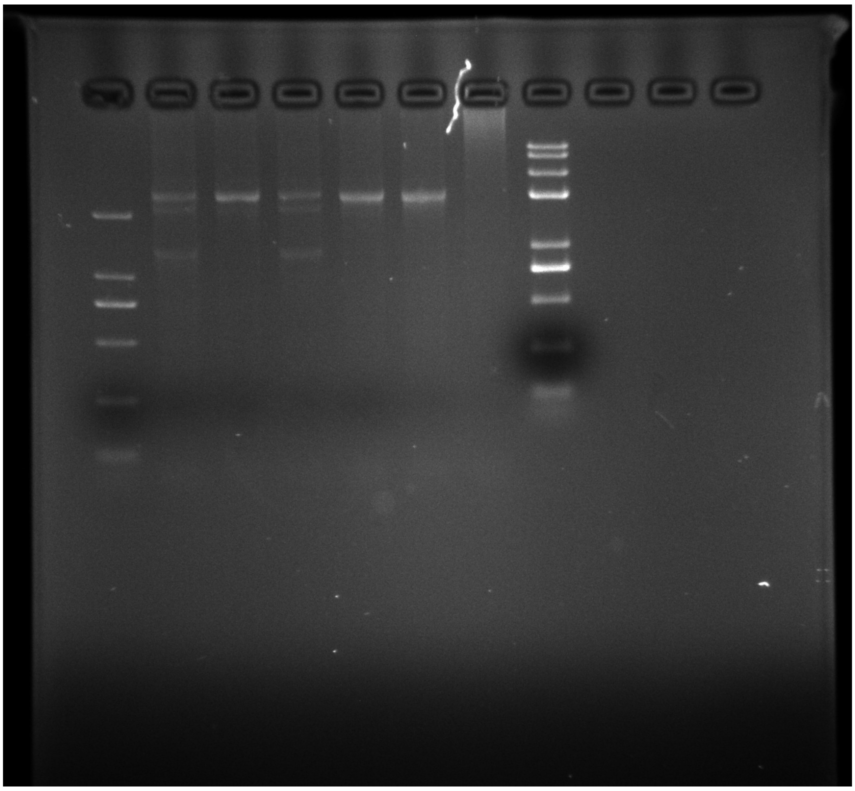

Supplementary Figure 1. Original gel image for long PCR. It was Marker2k, Ⅰ1, Ⅰ2, Ⅱ2, Ⅱ1, Control DNA, no template control, and irrelevant marker from left to right. The Marker2k included 2000bp, 1000bp, 750bp, 500bp, 250bp and 100bp in the line from top to bottom. Ⅰ1 was the father, Ⅰ2 was the mother, Ⅱ2 was the proband, Ⅱ1 was the sister. Control DNA was from an irrelative and unaffect individual. Two fragments were obtained (2,747 bp and 1,367 bp) from the patient (Ⅱ 2) and the father (Ⅰ 1).
